# Conjoint expression and purification strategy for acquiring proteins with ultra-low DNA N^6^-methyladenine backgrounds in *Escherichia coli*

**DOI:** 10.1042/BSR20203769

**Published:** 2021-03-15

**Authors:** Zheng Chen, Yan Liu, Hailin Wang

**Affiliations:** 1 State Key Laboratory of Environmental Chemistry and Ecotoxicology, Research Center for Eco-Environmental Sciences, Chinese Academy of Sciences, Beijing, 100085, China; 2 College of Resources and Environment, University of Chinese Academy of Sciences, Beijing 100049, China; 3 Institute of Environment and Health, Jianghan University, Wuhan 430056, China

**Keywords:** λRed system, DNA N6-methyladenine, E. coli Rosetta (DE3), low background, UHPLC-MS/MS

## Abstract

DNA N^6^-methyladenine (6mA), a kind of DNA epigenetic modification, is widespread in eukaryotes and prokaryotes. An enzyme activity study coupled with 6mA detection using ultra-high-performance liquid chromatography-quadruple mass spectrometry (UHPLC-MS/MS) is commonly applied to investigate 6mA potentially related enzymes *in vitro*. However, the protein expressed in a common *Escherichia coli* (*E. coli*) strain shows an extremely high 6mA background due to minute co-purified bacterial DNA, though it has been purified to remove DNA using multiple strategies. Furthermore, as occupied by DNA with abundant 6mA, the activity of 6mA-related proteins will be influenced seriously. Here, to address this issue, we for the first time construct a derivative of *E. coli* Rosetta (DE3) via the λRed knockout system specifically for the expression of 6mA-related enzymes. The gene *dam* encoding the 6mA methyltransferase (MTase) is knocked out in the newly constructed strain named LAMBS (low adenine methylation background strain). Contrasting with *E. coli* Rosetta (DE3), LAMBS shows an ultra-low 6mA background on the genomic DNA when analyzed by UHPLC-MS/MS. We also demonstrate an integral strategy of protein purification, coupled with the application of LAMBS. As a result, the purified protein expressed in LAMBS exhibits an ultra-low 6mA background comparing with the one expressed in *E. coli* Rosetta (DE3). Our integral strategy of protein expression and purification will benefit the *in vitro* investigation and application of 6mA-related proteins from eukaryotes, although these proteins are elusive until now.

## Introduction

Widespread DNA N^6^-methyladenine (6mA) is one of the most important epigenetic modifications and may play a vital role in many biological processes, either in prokaryotes or in eukaryotes [1–6]. The methyl group is transferred from S-adenosylmethionine to the N-6 position of adenine (A) by a methyltransferase (MTase), generating 6mA at a specific site. In *Escherichia coli* (*E. coli*) K-12 MG1655, it has been well demonstrated that there are three primary 6mA motifs: 6mA at 5′-GA(N_6_)TC-3′ mediated by Dam, 6mA at 5′-AA(N_6_)CGTGC-3′ mediated by HsdM, and 6mA at 5′-ATGCA(N_6_)T-3′ site mediated by YhdJ [7–12]. However, largely due to the low-level presence of 6mA, the distribution and functions of 6mA in eukaryotic genomic DNA are still elusive [2]. As one of the most sensitive detection methods of DNA modifications, ultra-high-performance liquid chromatography-quadruple mass spectrometry (UHPLC-MS/MS) has been widely applied to the study of 6mA [13,14]. *In vitro* activity analysis of proteins coupled with UHPLC-MS/MS analysis is a common research strategy. An interesting protein (potential 6mA reader or writer) can be overexpressed in a common *E. coli* expression strain such as Rosetta (DE3). However, we find that the 6mA level on the genomic DNA of Rosetta (DE3) is pretty high, which results in a high 6mA background on the protein due to minute co-purified bacterial DNA, although it has been purified to remove DNA using multiple strategies of protein purification. Furthermore, as occupied by DNA with high 6mA backgrounds, the activity of 6mA-related proteins will be influenced seriously [15,16]. Until now, it is still a great challenge to acquire proteins with ultra-low 6mA backgrounds from a common *E. coli* expression strain. Notably, according to the genotype, the existing Dam^−^ *E. coli* strains are suitable for plasmid cloning rather than protein overexpression.

Therefore, to essentially reduce the 6mA backgrounds on proteins for *in vitro* study, we knocked out the gene *dam*, which encodes 6mA MTase in *E. coli* Rosetta (DE3) via the optimized λRed knockout system, deriving a new expression strain termed LAMBS (low adenine methylation background strain). Consequently, the 6mA level was significantly reduced in LAMBS relative to that in the parental strain. Subsequently, through the integrally combined strategy of expression and purification proposed in this work, the co-purified bacteria DNA and 6mA could be removed from target proteins as much as possible. The final purified target protein not only showed an ultra-low 6mA background but also showed a considerably high yield and purity for the actual application. Overall, in the present paper, we provide an integrated strategy for the expression and purification of proteins associated with research on 6mA.

## Materials and methods

### Construction of LAMBS

*E. coli* Rosetta (DE3) competent cells were purchased from Biomed (Beijing, China). *E. coli* Rosetta (DE3) was selected as the parental strain because tRNA of six rare codons (AUA, AGG, AGA, CUA, CCC, and GGA) are supplied by a plasmid (pRARE, chloramphenicol-resistant) (Novagen, WI, U.S.A.) to enhance the overexpression of eukaryotic proteins. The helper plasmids used in the λRed knockout system were gifts from Prof. Xiaoyun Liu (School of Basic Medical Sciences, Peking University Health Science Centre, Beijing, China) who also supplied the electroporation instrument (Bio-Rad Micropulser Electroporator 1652100, U.S.A.). The medium for all culture experiments was Lysogeny Broth (LB) (5 g of yeast extract, 10 g of tryptone, and 10 g of sodium chloride per 1 l medium, pH 7.4), and LB solid medium (LB plate) was prepared by adding 2% agar (w/v) into the LB medium. The relevant working concentration of ampicillin, chloramphenicol, and kanamycin was 100 μg/ml, 25 μg/ml, and 30 μg/ml respectively. The antibiotics, l-arabinose, and materials for LB preparation were all purchased from Solarbio (Beijing, China). The Q5 high-fidelity polymerase for PCR was purchased from New England Biolabs (Ipswich, MA, U.S.A.). The primers were synthesized by Sangon (Shanghai, Beijing).

The application of the λRed knockout system was as described previously [17–20]. Three plasmids are needed for an integral process of gene knockout. Plasmid pKD13 is used as a template to prepare an antibiotic resistance marker. Under the induction of l-arabinose, Plasmid pKD46 expresses three enzymes involved in gene knockout. Plasmid pCP20 is induced by temperature to express enzymes that can eliminate antibiotic resistance marker from genomic DNA.

To make this strain electrocompetent, Rosetta (DE3) cells were transformed with pKD46 and then plated on LB plates supplemented with chloramphenicol and ampicillin. After overnight culture at 30°C, one new clone was inoculated into 10 ml LB medium with chloramphenicol, ampicillin, and 20 mM l-arabinose. When the OD_600_ reached approximately 0.6, the bacteria cells were washed three times with 40 ml of pre-cooled 10% glycerol. After resuspension, the newly prepared electrocompetent cells were stored at −80°C. A strategy of overlapping PCR was utilized to create *kan* fragments with homologous arms (500 bp) to *dam* upstream and downstream. In brief, three independent PCRs were required. One was to yield the *kan* cassette using pKD13 as a template and P5-P6 as primers. The other two were to create fragments upstream and downstream *dam*, which had 25 bp homologous arms to the *kan* cassette. In the two reactions, P1-P2 and P3-P4 were used as primers separately, and the genomic DNA of Rosetta (DE3) was used as a template. After being purified using agarose gel electrophoresis, these three independent PCR products were mixed at a mole ratio of 1:1:1. In the overlap PCR, the mixture was then used as a template, and P1-P4 were used as the primers (Table 1).

**Table 1:** DNA sequences of primers for overlap PCR

Primers	DNA sequence
P1	5′ – ACTGAACCAGCTGCTCCT – 3′
P2	5′ – CGACGGATCCCCGGAATTAATTCTGCTGACTAACTAATTACACCTTC – 3′
P3	5′ – CAGCCTACACAATCGCTCAAGTTCTCAAGGAGAAGCGGATGAAACA – 3′
P4	5′ – GACGTACTTCGCGCAGTTTA – 3′
P5	5′ – AGAATTAATTCCGGGGATCCGTC – 3′
P6	5′ – TCTTGAGCGATTGTGTAGGCTG – 3′

After gel-purification, 500 ng of this overlapping PCR product was transformed into 50 μl of prepared Rosetta (DE3) electrocompetent cells using electroporation. After incubation in LB at 30°C for 2 h, the electroshocked cells were spread on LB plates to select ampicillin and kanamycin-resistant transformants at 37°C. Positive clones were verified by PCR using primers as described previously (Supplementary Table S1). A further culture was conducted at 37°C to eliminate the pKD46 plasmids. Finally, the new strain Rosetta (DE3) (Δ*dam*::*kan*) was named LAMBS. pCP20 was then transformed into electrocompetent cells of LAMBS and grown at 30°C to select ampicillin-resistant transformants. Therefore, the elimination of pCP20 and *kan* inserted in the genomic DNA was accomplished at the same time through a further overnight growth at 42°C.

### Determination of specific growth rate

As described previously, specific growth rates of both strains in the log phase were calculated using OD_600_ as the parameter. First, measurements of OD_600_ were performed using a Varioskan Flash Microplate Reader (Thermo, U.S.A.). Both strains were cultured at 37°C and 200 rpm to an OD_600_ ≈ 0.6 and diluted with LB to an OD_600_ ≈ 0.02 (1:2000 v/v). Then 200 μl per well of those dilutions was added into a 96-well plate in triplicate for a continuous bacteria culture at 37°C and 200 rpm. The OD_600_ was measured three times every 2 h from the initial culture. The growth of both strains was supplemented with 25 μg/ml chloramphenicol. Therefore, the growth curves over 24 h at 37°C could be plotted. Second, the specific growth rate (*μ*, where *t* is time) and doubling time (*T_s_*) were calculated using the formula (1) and (2), respectively.

(1) $$\mu =\Delta ln\;OD_{600}/\Delta t$$

(2) $$T_{s}=\ln\;2/\mu$$

### Protein expression

We used a previously constructed and stored plasmid to test the performance of target protein expression and purification in LAMBS cells. The backbone of this plasmid (pMAL-c5X-Thrombin) is pMAL-c5X (#N8108, New England Biolabs) with a maltose-binding protein (MBP) tag. And the Factor Xa site is replaced with a thrombin cleavage site. The gene (GeneID: 29104) encoding the target protein (N6AMT1, NP_037372.4) for performance testing had been cloned into it (between Nco I and BamH I) previously using EasyGeno Assembly Cloning Kit purchased from Tiangen (Beijing, China) (Supplementary Figure S1). In brief, 10 ng of this plasmid was transformed into 100 μl of LAMBS competent cells that were prepared by the calcium chloride method [21]. Positive clones were selected by chloramphenicol and ampicillin resistance at 37°C through overnight culture on LB plates. One clone was directly inoculated into 50 ml of LB medium following overnight growth at 37°C. As an initial inoculum, 12.5 ml of this overnight culture was added to 500 ml LB medium for further culture. While the OD_600_ reached ∼0.6, IPTG was added to the broth to a final concentration of 0.2 mM. After overnight induction at 14°C, a total of 2 l of broth was centrifuged for 3 min at 7500*×****g*** and 4°C. The cell pellet was then immediately resuspended in 30 ml of lysis buffer and was lysed using an Ultra High-Pressure Cell Crusher (JNBIO, Guangzhou, China). During the procedure of protein expression and purification, all the buffers (Supplementary Table S2) were pre-cooled and all the operations of centrifuges were carried out at 4°C.

### Polyethyleneimine precipitation

After cell lysis, polyethyleneimine (PEI, MW ∼25000, Sigma, U.S.A.) precipitation was performed [22]. The supernatant was collected in a conical flask after the crude lysed cell suspension had been centrifuged for 15 min at 7500*×****g***. Five percent PEI (m/v) was then slowly added in the supernatant to a final concentration of 0.5%, with constant stirring for 30 min. Following this, the mixture was centrifuged 20 min at 7500*×****g*** and the supernatant was then placed in a new conical flask.

### Ammonium sulfate precipitation

A saturated ammonium sulfate (AS) solution prepared in advance was carefully added to the newly collected supernatant drop by drop to a final 60% saturation with constant stirring for 1 h. After centrifugation for 20 min at 7500*×****g***, the target protein was precipitated and washed two times with lysis buffer containing 60% saturated AS. Finally, the target protein containing precipitate was dissolved in 30 ml of HIC binding buffer.

### Chromatographic purification

Chromatographic purification was performed using an AKTA Purifier UPC10 System (GE Healthcare, Uppsala, Sweden). The protein solution and all buffers were prefiltered through a 0.22-μm filter membrane and then stored at 4°C. The columns for hydrophobic interaction chromatography (HIC, Phenyl Sepharose 6 FF, 17-0965-10), MBP affinity chromatography (MBP, MBPtrap HP, 28-9187-78), and ion-exchange chromatography (MonoQ, 10/100 GL, 17-5167-01) were purchased from GE Healthcare. First, the protein solution was loaded on the HIC column and then eluted using a concentration gradient of (NH4)_2_SO_4_ (from 1 to 0 M). Next, the elution fraction of HIC including target protein was loaded on an MBP column and eluted by buffer with 1 mM maltose. The target protein was then dialyzed so that the cleavage at the thrombin site (2 U/1 mg protein) could be carried out overnight at 4°C. Finally, the mixture of thrombin digested products was loaded on a MonoQ column and eluted using a concentration gradient of NaCl (from 0.5 to 1 M). In every step of purification, 20 μl of each solution was subjected to 10% SDS/PAGE to confirm the presence of the target protein and then quantified using a bovine serum albumin (BSA) standard on ImageJ software.

### UHPLC-MS/MS analysis

Newly picked clones from both strains were respectively inoculated in 5 ml of fresh LB medium and cultured overnight at 37°C. Subsequently, 2 ml of LAMBS and Rosetta (DE3) cells was centrifuged and harvested for the extraction of genomic DNAs using a TIANamp Bacteria DNA Kit purchased from Tiangen (Beijing, China). Besides, 3 μg of purified protein was digested with 0.5 U Proteinase K (Thermo, U.S.A.) for 1 h so that the co-purified DNA could be recovered by phenol-chloroform extraction. For the digestion of DNA, snake venom phosphodiesterase I (SVP) was obtained from Worthington Biochemical Corporation (Lakewood, U.S.A.), and calf intestinal alkaline phosphatase (CIP) and deoxyribonuclease I (DNase I) were ordered from New England Biolabs. The quantification of DNA was performed using a NanoDrop 2000 (Thermo, U.S.A.). Every 1 μg of genomic DNA and the entire co-purified DNA were digested to 2′-deoxynucleosides by an enzyme mixture of 1 U CIP, 0.5 U DNase I, and 0.003 U SVP at 37°C for 6 h. The digested DNA samples were filtered by ultrafiltration and then were subjected to UHPLC-MS/MS analysis. An Agilent 1290 II UHPLC system coupled with ESI-triple quadrupole mass spectrometers 6470 and 6410 (Agilent Technologies, U.S.A.) was respectively applied to the detection of 6mA (m/z 266–150) and DNA Cytosine (dC) (m/z 228–112). The conditions for chromatography and mass spectrometry were as we reported previously [13,14,16,23].

## Results

### Construction of LAMBS via an optimized λRed knockout system

Aiming to eliminate the 6mA background as much as possible on proteins expressed and purified from *E. coli*, we utilized the λRed system to knock out the gene *dam*, which encodes the 6mA MTase. To prepare the 2400 bp substrate DNA, overlap PCR was applied to the ligation of three DNA fragments and amplification *in vitro* [24,25]. Analysis by the agarose gel electrophoresis, the results showed that the yield and specificity of target overlap PCR products were high enough for the next application (Supplementary Figure S2A). Then, 500 ng of substrate DNA was transformed into Rosetta (DE3) cells, and after overnight culture, hundreds of clones were observed on LB plates, showing a respectable transformation rate. Furthermore, ten clones were randomly picked for further verification of a correct *kan* insertion using bacterial colony PCR. As a result, all ten clones were confirmed to be positive (Supplementary Figure S2A). Subsequently, through the transformation of pCP20 and further resistance selection, we succeeded in excising the *kan* gene in the genomic DNA of LAMBS, yielding the final strain (Supplementary Figure S2B).

### Determination of specific growth rate

To guide the bacterial culture of the newly constructed strain, it was necessary to determine the specific growth rate. As reported previously, the growth curves were plotted by the measured OD_600_ (Supplementary Figure S3). It could be observed that LAMBS cells had a very long lag phase (∼6 h) before entering the log phase. when LAMBS cells shifted into the log phase, Rosetta (DE3) cells had already entered the stable phase. Moreover, the OD_600_ values of LAMBS were lower than that of Rosetta (DE3) throughout the growth experiment (Supplementary Figure S3). Following the identification of the exponential phase, the specific growth rate of each strain was calculated. As a result, the specific growth rate of Rosetta (DE3) (*μ* = 1.37 h^−1^) was much higher than that of LAMBS (*μ* = 0.34 h^−1^). And LAMBS strain showed a more prolonged doubling time (*T_s_* = 2.03 h) than Rosetta (DE3) (*T_s_* = 0.51 h).

### Analysis of the 6mA background in LAMBS

**Figure 1:**
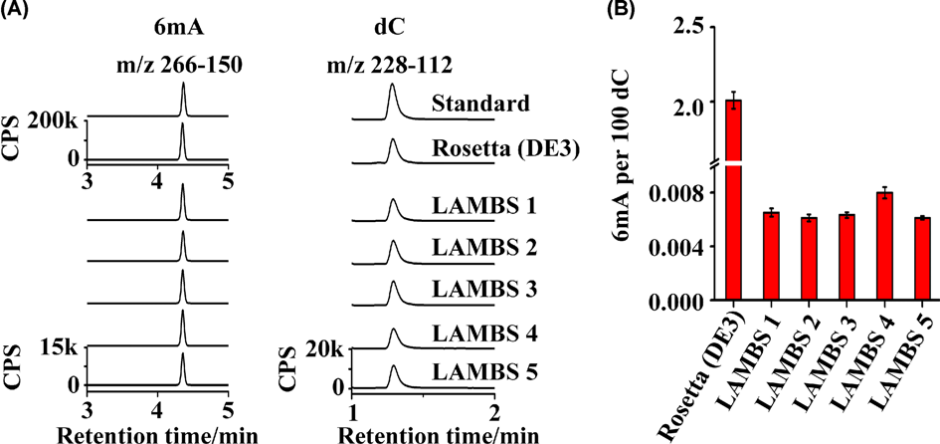
UHPLC-MS/MS analysis of the 6mA background in LAMBS (**A**) UHPLC-MS/MS chromatograms of 6mA (m/z 266–150) and dC (m/z 228–112). The genomic DNA of LAMBS and Rosetta (DE3) was extracted and then digested to 2′-deoxynucleosides for the detection of 6mA and dC using UHPLC-MS/MS. (**B**) Quantification of 6mA backgrounds in five independent clones of LAMBS with Rosetta (DE3) as a control.

As discussed above, the high 6mA background in a purified protein is often from the co-purified genomic DNA of *E. coli*. Therefore, before the practical application of LAMBS, it was important to study how much the 6mA background was reduced on the genomic DNA of LAMBS, in which the *dam* gene had been knocked out in LAMBS. We utilized UHPLC-MS/MS to analyze the difference of the 6mA background between LAMBS and Rosetta (DE3) cells (Figure 1A). To minimize influences from the experimental operation, we took five independent clones of LAMBS to analyze the changes in the 6mA background with Rosetta (DE3) as a control. Our results showed that the 6mA level (6mA/dC) was ∼2.0% in Rosetta (DE3) and ∼0.008% in LAMBS respectively, where a reduction by three orders of magnitude was observed (Figure 1B).

### Protein expression and purification in LAMBS

**Figure 2:**
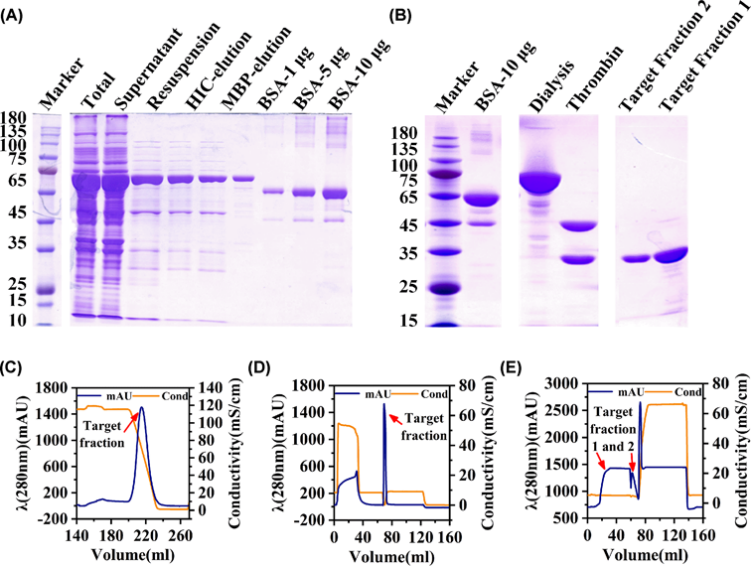
SDS/PAGE gel analysis of the target fraction and the chromatograms of protein purification (**A**) SDS/PAGE analysis of 20 μl of total protein, supernatant, resuspension after AS precipitation, HIC, and MBP target fraction with BSA as a standard. The lanes were from the same gel, and the marker lane was cut and moved to the left of the image. (**B**) SDS/PAGE analysis of the target protein after dialysis, digestion with thrombin, and MonoQ chromatography. (**C**–**E**) Chromatograms of the target protein purification using HIC, MBP, and MonoQ chromatography respectively. The lanes were from the same gel and were cut and moved horizontally.

A practical application test of LAMBS was carried out. As shown by SDS/PAGE (Figure 2A), almost all the target protein was contained in the supernatant after cell lysis and was well purified through PEI and AS precipitation though ∼50% of that was lost. The target protein was then purified by HIC chromatography, and ∼30 ml eluted fraction (Figure 2C) was further purified and concentrated to ∼5 ml by MBP affinity chromatography (Figure 2D). According to our gel results, the purity of the target protein was over 85% and the yield of it was ∼2.5 mg per 2 l of LAMBS cells through these purification steps. After dialysis, the target protein was thoroughly cleaved at the thrombin site (Figure 2B) and then separated from the recombinant MBP tags by MonoQ chromatography (Figure 2E). As a result, the purity of the target protein was further improved to over 90%. After being quantified by ImageJ with BSA as a standard, the concentration of the target protein was determined to be 0.2 μg/μl in the MBP elution and 0.1 μg/μl in the MonoQ target fraction 2, respectively.

### Analysis of the 6mA background on the target protein

**Figure 3:**
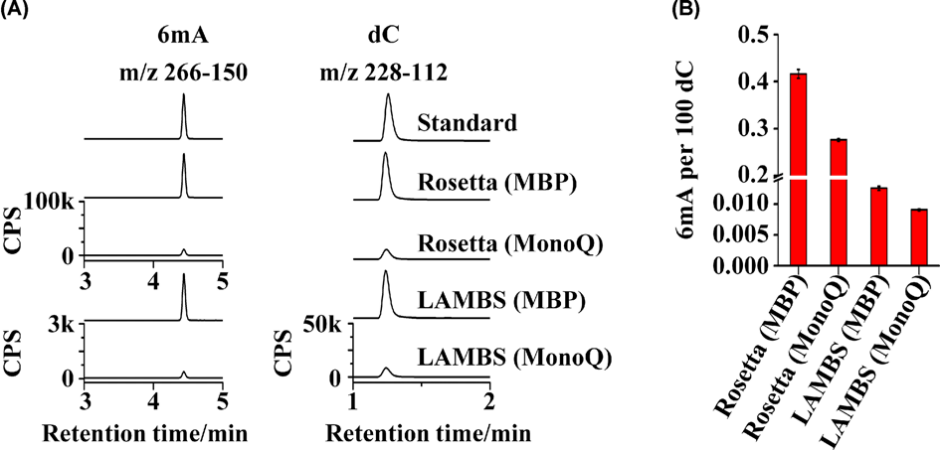
UHPLC-MS/MS analysis of the 6mA background in the purified target protein (**A**) UHPLC-MS/MS chromatograms of 6mA (m/z 266–150) and dC (m/z 228–112). The DNA co-purified with the target protein was extracted and then digested to 2′-deoxynucleosides for the detection of 6mA and dC using UHPLC-MS/MS. (**B**) Quantification of the 6mA background in the purified target protein after the MBP affinity chromatography and after the MonoQ chromatography.

Above all, the final question was whether the 6mA background on the target protein was drastically reduced as expected, even though the LAMBS exhibited excellent performance for protein expression and purification. We took 5 μg of the same target protein stored previously as a control, as it was acquired by the same expression and purification methods in Rosetta (DE3) cells (data not shown). The abundance of dC represents the amount of DNA. It was obvious that the DNA co-purified with protein could be removed efficiently through MonoQ chromatography (Figure 3A).

Besides, the 6mA abundance of the target protein from LAMBS was much lower than that from Rosetta (DE3), whether purified by MonoQ chromatography or not (Figure 3A). Moreover, through the same purification by MonoQ chromatography, the 6mA background of the target protein from LAMBS decreased as much as 2 orders of magnitude (from ∼0.28 to ∼0.008%) compared with that from Rosetta (DE3) cells (Figure 3B). Additionally, the 6mA background of the target protein from LAMBS was even much lower than that from Rosetta (DE3), which was purified through MonoQ chromatography.

## Discussion

As reported previously, the λRed system displays high recombination frequency, though the homology arms to a targeted insert site as short as 35 bp. However, short homologous arms are easily degraded by exonucleases that are incompletely suppressed [26]. Therefore, we increased the length of the homology arms to 500 bp and realized a high-efficient gene knockout. After knockout of the gene *dam*, the OD_600_ of LAMBS over 24 h was measured to determine the specific growth rate. In the parental strain Rosetta (DE3), Dcm (DNA cytosine methyltransferase) is deficient, which mediates the generation of C^5^-methylcytosine (5mC) at 5′-C(N_5_)C(A/T)GG-3′ sites [12]. Dcm is also associated with the very short patch mismatch (VSP) repair [27,28]. Even though the Dam or Dcm is non-essential for the survival of *E. coli*, the deficiency of both methylases could result in increased levels of DNA double-strand breaks and mutation rates [29,30]. The DNA repair system depending on DNA methylation at specific sites can not work, which may result in a reduction in the specific growth rate [31]. Therefore, LAMBS grows much slower than Rosetta (DE3). Therefore, a prolonged culture is necessary for obtaining enough LAMBS cells when applied to protein expression. Subsequently, an analysis of the 6mA background in LAMBS was performed using UHPLC-MS/MS. Contrasting with Rosetta (DE3), a reduction in three orders of magnitude in the 6mA level was observed in our result. According to the genotype, HsdM was deficient in LAMBS, which is inherited from Rosetta (DE3), and the gene *dam* was knocked out. Therefore, the source of residual 6mA (∼0.008%, 6mA/dC) should be primarily mediated by YhdJ.

LAMBS was then applied to protein expression. Overnight culture and induction were performed to improve the expression of the recombinant protein. The problem is to choose a properly combined strategy for protein purification, which should isolate DNA from the target protein as much as possible and should save time to avoid protein inactivation. Therefore, we propose a combined and sequential strategy for protein purification, which includes PEI precipitation, AS precipitation, HIC chromatography, MBP chromatography, and MonoQ chromatography. Protein is stable in PEI and AS precipitation and could be purified to remove much DNA. Furthermore, through the preparation of proper buffers, HIC chromatography, MBP chromatography, and MonoQ chromatography can be performed one by one to save time and also to remove DNA [32–37]. This strategy results in enough high yield and purity of the target protein. And in principle, this strategy has universality for the purification of protein with an MBP tag. When analyzed using UHPLC-MS/MS, the 6mA background on the finally purified target protein from LAMBS is ∼8/10^5^ (6mA/dC). The 6mA level on the eukaryotic genomic DNA is ∼1/10^7^ (6mA/dA) [16]. This indicates that the 6mA background of the protein obtained by our conjoint expression and purification strategy is reduced much close to the 6mA level in eukaryotes. And it is not bound by excessive bacterial DNA that contains an abundance of 6mA.

## Conclusion

In summary, through the knockout of the gene *dam* via the λRed knockout system, we developed a new expression strain LAMBS derived from Rosetta (DE3) cells and proposed a combined strategy for applying LAMBS to practical protein expression and purification. As analyzed by UHPLC-MS/MS, the 6mA background of the target protein from LAMBS was reduced greatly relative to that from Rosetta (DE3), a common expression strain. Furthermore, the ultra-low 6mA background on the purified protein (∼8/10^5^, 6mA/dC) also indicated that LAMBS would be a proper tool strain for the *in vitro* study of 6mA-related proteins.

## Supplementary Material

Supplementary Figures S1-S3 and Tables S1-S2

## Data Availability

All data included in the present study are available by contacting the corresponding author.

## Abbreviations

BSA: bovine serum albumin
CIP: calf intestinal alkaline phosphatase
dC: DNA cytosine
Dcm: DNA cytosine methyltransferase
DNase I: deoxyribonuclease I
HIC: hydrophobic interaction chromatography
IPTG: isopropyl-β-d-thiogalactoside
LAMBS: low adenine methylation background strain
LB: lysogeny broth
MBP: maltose-binding protein
MTase: methyltransferase
PEI: polyethyleneimine
SVP: snake venom phosphodiesterase I
UHPLC-MS/MS: ultra-high-performance liquid chromatography-quadruple mass spectrometry
5mC: C^5^-methylcytosine
6mA: N^6^-methyladenine
