# Supplementary material for: Conjoint expression and purification strategy for acquiring proteins with ultra-low DNA N6-methyladenine backgrounds in Escherichia coli
Source: Biosci Rep. 2021 Mar 15;41(3):BSR20203769. doi: 10.1042/BSR20203769 (PMC7960888; doi:10.1042/BSR20203769)
Supplement: Supplementary Figures S1-S3 and Tables S1-S2 [file BSR-2020-3769_supp.pdf]

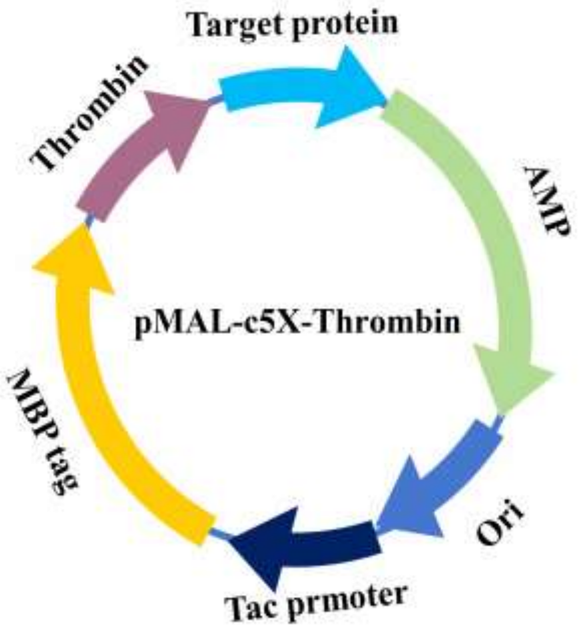

Figure S1.Sketch map of the plasmid used for the target protein expression.

The gene encoding the target protein was inserted behind the thrombin site. Themolecular weight of the target protein is about 30 kDa. The length of the target protein with an MBP tag is about 73 kDa.

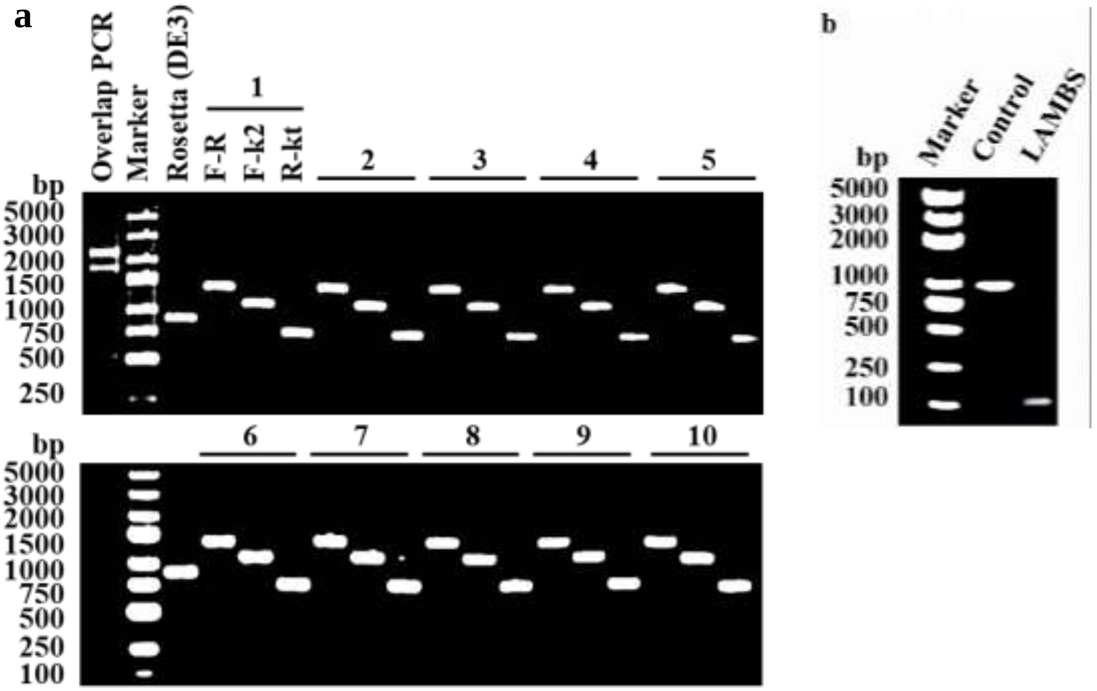

Figure S2. Agarose gel electrophoresis analysis of overlap PCR and bacteria PCR.

With Rosetta (DE3) as a control, 10 independent clones were randomly picked for further verification of gene knockout using F-R, F-K2 and R-Kt as primers. **a.** Overlap PCR for the preparation of substrate DNA (the first lane). The length of target overlap PCR products is 2302 bp. Bacteria PCR for the identification of *kan* insertion.The F-R length of Rosetta (DE3) is 834bp. The F-R length of LAMBS is 1353 bp, while the length of F-k2 and R-kt is 1079 bp and 710 bp respectively. **b.** Bacteria PCR for the verification of *kan*elimination. F-R were used as primers. The length of Rosetta (DE3)is 834 bp and that of LAMBSis about 120 bp.

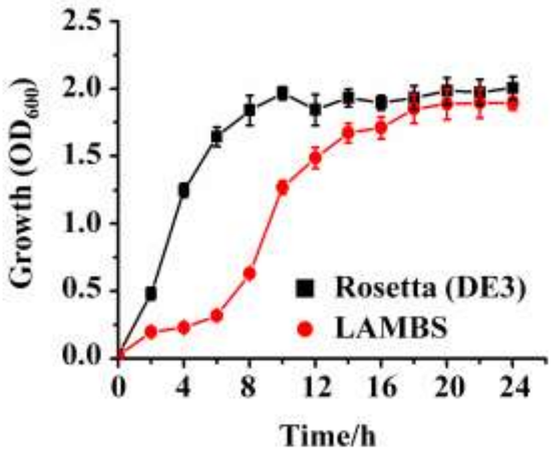

Figure S3. The growth curves (OD_{600}) of LAMBS and Rosetta (DE3).

Both tow strains were simultaneously cultured at 37 °C from an initial OD_{600} of ≈ 0.02 at 0 h until 24 h, and the OD_{600} was measured every 2 h.

Table S1. Primers for PCR identification of the *dam*gene knockout

Primer	Sequence
F	5’ – CTGCTTGATGATATTAAACGGCATTTGC – 3’
R	5’ – CTTATACTGCGTCGAACTTTGACGAC – 3’
K2	5’ – CGGTGCCCTGAATGAACTGC – 3’
Kt	5’ – CGGCCACAGTCGATGAATCC – 3’

Table S2. Buffers for protein purification

Buffer	Preparation
Lysis buffer	pH 7.6, 50 mM Tris-HCl, 100 mM NaCl
HIC Binding buffer	pH 7.6, 50 mM Tris-HCl, 100 mM NaCl and 1 M $(NH_4)_2SO_4$
HIC Elution buffer	pH 7.6, 50 mM Tris-HCl, 100 mM NaCl
MBP Binding buffer	pH 7.6, 50 mM Tris-HCl, 100 mM NaCl
MBP Elution buffer	pH 7.6, 50 mM Tris-HCl, 100 mM NaCl and 1mM maltose
MonoQ Binding buffer	pH 8.0, 50 mM Tris-HCl, 100 mM NaCl
MonoQ Elution buffer	pH 8.0, 50 mM Tris-HCl, 1 M NaCl
Dialysis buffer	pH 8.0, 50 mM Tris-HCl, 100 mM NaCl

Notes: Each buffer contains 1 mM PMSF, 1mM DTT and10% glycerol
